# Supplementary material for: Multi‐dataset identification of innovative feature genes and molecular mechanisms in keratoconus
Source: J Cell Mol Med. 2024 Sep 19;28(18):e70079. doi: 10.1111/jcmm.70079 (PMC11412914; doi:10.1111/jcmm.70079)
Supplement: Supplementary file 2 — Material S2. [file JCMM-28-e70079-s002.zip › 7.GSEA analysis/1.ARL11/KEGG_GLYCEROLIPID_METABOLISM.html]

Details for gene set KEGG\_GLYCEROLIPID\_METABOLISM[GSEA]

|  || Dataset | ARL11.group.cls#h\_versus\_l |
| Phenotype | group.cls#h\_versus\_l |
| Upregulated in class | h |
| GeneSet | KEGG\_GLYCEROLIPID\_METABOLISM |
| Enrichment Score (ES) | 0.27189863 |
| Normalized Enrichment Score (NES) | 0.95484364 |
| Nominal p-value | 0.50094515 |
| FDR q-value | 1.0 |
| FWER p-Value | 1.0 |
Table: GSEA Results Summary

  

Fig 1: Enrichment plot: KEGG\_GLYCEROLIPID\_METABOLISM      
 Profile of the Running ES Score & Positions of GeneSet Members on the Rank Ordered List

  

| SYMBOL | TITLE | RANK IN GENE LIST | RANK METRIC SCORE | RUNNING ES | CORE ENRICHMENT || 1 | GPAM | na | 898 | 0.401 | 0.0455 | Yes |
| 2 | GK2 | na | 1619 | 0.326 | 0.0829 | Yes |
| 3 | CEL | na | 2807 | 0.250 | 0.0932 | Yes |
| 4 | DGKE | na | 3504 | 0.219 | 0.1122 | Yes |
| 5 | AGPAT4 | na | 3585 | 0.216 | 0.1484 | Yes |
| 6 | PNLIPRP2 | na | 3781 | 0.209 | 0.1799 | Yes |
| 7 | GLYCTK | na | 3983 | 0.201 | 0.2100 | Yes |
| 8 | DGKG | na | 6475 | 0.125 | 0.1604 | Yes |
| 9 | DGKD | na | 6734 | 0.118 | 0.1740 | Yes |
| 10 | PNPLA3 | na | 7025 | 0.111 | 0.1854 | Yes |
| 11 | DGKA | na | 7100 | 0.110 | 0.2029 | Yes |
| 12 | MBOAT2 | na | 7404 | 0.104 | 0.2126 | Yes |
| 13 | DGKQ | na | 7475 | 0.102 | 0.2288 | Yes |
| 14 | LIPF | na | 7863 | 0.095 | 0.2346 | Yes |
| 15 | DGKZ | na | 8028 | 0.092 | 0.2463 | Yes |
| 16 | LIPC | na | 8203 | 0.088 | 0.2570 | Yes |
| 17 | DGKB | na | 8228 | 0.088 | 0.2719 | Yes |
| 18 | LCLAT1 | na | 8846 | 0.077 | 0.2679 | No |
| 19 | AGPAT3 | na | 10303 | 0.056 | 0.2359 | No |
| 20 | AKR1A1 | na | 10312 | 0.056 | 0.2457 | No |
| 21 | DGKH | na | 10341 | 0.056 | 0.2548 | No |
| 22 | ALDH7A1 | na | 10913 | 0.048 | 0.2470 | No |
| 23 | DGAT1 | na | 11620 | 0.040 | 0.2338 | No |
| 24 | AGPAT2 | na | 12559 | 0.031 | 0.2122 | No |
| 25 | AGK | na | 12814 | 0.029 | 0.2100 | No |
| 26 | MGLL | na | 12864 | 0.028 | 0.2136 | No |
| 27 | ALDH3A2 | na | 13283 | 0.025 | 0.2059 | No |
| 28 | DGAT2 | na | 14557 | 0.015 | 0.1718 | No |
| 29 | DGKI | na | 15757 | 0.005 | 0.1382 | No |
| 30 | MBOAT1 | na | 16198 | 0.002 | 0.1259 | No |
| 31 | AGPAT1 | na | 16949 | -0.003 | 0.1048 | No |
| 32 | GPAT2 | na | 23247 | -0.053 | -0.0673 | No |
| 33 | GLA | na | 23413 | -0.055 | -0.0623 | No |
| 34 | ALDH1B1 | na | 25126 | -0.078 | -0.0978 | No |
| 35 | ALDH9A1 | na | 25157 | -0.078 | -0.0847 | No |
| 36 | LIPG | na | 25530 | -0.083 | -0.0806 | No |
| 37 | PNLIP | na | 27920 | -0.129 | -0.1265 | No |
| 38 | GK | na | 30263 | -0.196 | -0.1591 | No |
| 39 | AKR1B1 | na | 30604 | -0.208 | -0.1319 | No |
| 40 | ALDH2 | na | 31150 | -0.226 | -0.1073 | No |
| 41 | PNLIPRP1 | na | 31630 | -0.245 | -0.0775 | No |
| 42 | LPL | na | 33967 | -0.415 | -0.0708 | No |
| 43 | AWAT2 | na | 34464 | -0.520 | 0.0076 | No |
Table: GSEA details [plain text format]

  

Fig 2: KEGG\_GLYCEROLIPID\_METABOLISM      
 Blue-Pink O' Gram in the Space of the Analyzed GeneSet

  

Fig 3: KEGG\_GLYCEROLIPID\_METABOLISM: Random ES distribution      
 Gene set null distribution of ES for **KEGG\_GLYCEROLIPID\_METABOLISM**
